# NOD2 Variants and Duration of Hospitalization After Endoscopic Treatment of Biliary Strictures Following Liver Transplantation: A Cutoff-Dependent Association

**DOI:** 10.3390/jcm15197470

**Published:** 2026-09-25

**Authors:** Muhammed Yalçın, Nevra Yalçın, Muhsin Murat Muhip Harputluoglu, Baris Otlu

**Affiliations:** 1 Division of Gastroenterology, Department of Internal Medicine, Ankara Etlik City Hospital, 06170 Ankara, Türkiye; 2 Division of Medical Oncology, Department of Internal Medicine, Ankara Etlik City Hospital, 06170 Ankara, Türkiye; 3 Department of Gastroenterology, Faculty of Medicine, Inonu University, 44280 Malatya, Türkiye; 4 Department of Medical Microbiology, Faculty of Medicine, Inonu University, 44280 Malatya, Türkiye

**Keywords:** biliary stricture, ERCP, liver transplantation, NOD2

## Abstract

**Background/Objectives:** Nucleotide-binding oligomerization domain containing 2 (NOD2) variants are an important genetic risk factor that can affect the course of a disease via interaction between the gut and liver in patients with liver diseases. In this study, we investigated the effects of NOD2 variants on the hospital stay time in patients who underwent endoscopic treatment for biliary strictures after living donor liver transplantation (LDLT). **Methods:** Age; gender; time to endoscopic retrograde cholangiopancreatography (ERCP) after transplantation; ERCP findings; and hospitalization time of patients who underwent ERCP for biliary strictures after LDLT were recorded. The two hundred patients included in the study were divided into two groups: Group 1, discharged during the first 24 h after ERCP, and Group 2, discharged after the first 24 h following ERCP. The frequency of three NOD2 variants was investigated for the two groups. **Results:** The number of patients whose strictures could not be passed with endoscopic intervention in Group 2 was significantly higher than the other group (34% vs. 0%, *p* < 0.0001). In addition, p.R702W and 1007fs mutation frequencies in Group 2 were significantly higher than in Group 1 (10% vs. 0%, *p* = 0.002, and 14% vs. 3%, *p* = 0.011, respectively). In a Firth (bias-reduced) multivariable logistic regression including all 200 patients and adjusting for stricture-passage failure, age, CRP, and IL-6, NOD2 variant carriage remained associated with prolonged hospitalization after adjustment for the measured covariates (OR: 14.55; 95% CI: 3.06–69.04; *p* < 0.001), a finding corroborated by a sensitivity analysis restricted to patients with successful stricture passage (OR: 11.02; 95% CI: 2.24–54.08; *p* = 0.003). This association was specific to the 24 h discharge threshold used to define the primary outcome: NOD2 variant carriage was not significantly associated with length of stay when analyzed continuously (fold-change 1.27; *p* = 0.182) and lost statistical significance at longer discharge thresholds (>48 h; *p* = 0.075; >72 h; *p* = 0.235). **Conclusions:** Our findings suggest that there may be a relationship between NOD2 variants, especially the p.R702W variant, and length of hospital stay in patients undergoing ERCP after LDLT when defined by the 24 h discharge threshold used here. This cutoff-dependent association requires confirmation using continuous length-of-stay outcomes and prespecified thresholds in larger, multicenter cohorts.

## 1. Introduction

Donor age, prolonged cold and warm ischemia time, surgical techniques, and development of hepatic artery thrombosis are well-known risk factors for biliary stricture formation after living donor liver transplantation (LDLT) [1]. Although significant improvements have been made to overcome these problems, biliary strictures are still one of the most important causes of morbidity and mortality after LDLT.

The gut and liver share essential anatomic and functional connections, and the interaction between the gut and liver has been implicated in the development of several liver diseases, such as biliary inflammatory diseases. The pathogenesis underlying these processes remains unknown [2]. Nucleotide-binding oligomerization domain containing 2 (NOD2) is a component of the innate immune system and expressed in Paneth cells of the intestinal epithelium, as well as hepatocytes, macrophages, and dendritic cells [3,4,5]. NOD2 is a NOD-like receptor family member that senses and responds to bacterial wall peptides [6]. The protein facilitates a balance between the host immune system and intestinal flora elements [7], regulates the secretion of several anti-bacterial compounds, augments intracellular bacterial killing [8], and induces a variety of cytokines and chemokines by activating NF-κB and mitogen-activated protein kinases [9]. Genetic variants of NOD2 are associated with Crohn’s disease [10,11]. In a previous study, we demonstrated a strong association between a NOD2 variant and spontaneous bacterial peritonitis in patients with cirrhosis [12]. Furthermore, Janse et al. [13] reported that the R702W NOD2 variant is associated with an increased risk of bacterial infections after orthotopic liver transplantation. In another recent study, an association between NOD2 gene variants and secondary sclerosing cholangitis was reported in critically ill patients [14].

In this study we investigated the effects of some NOD2 variants (p.G908R, p.R702W, and 1007fs) on the length of hospital stay and outcomes among patients who underwent endoscopic treatment for biliary strictures after LDLT.

## 2. Materials and Methods

### 2.1. Determination of Patient Number in Groups

To determine the sample size, a previous publication investigating the frequency of NOD2 variants in liver transplantation was used [13]. Using a reported NOD2 variant frequency of 13.6% [13] in transplant recipients, the sample size required to estimate this prevalence with a 95% confidence interval and a margin of error of ±4.76% was calculated to be 199. This calculation addressed only the precision of an overall prevalence estimate; a separate a priori power calculation for the between-group comparison of NOD2 variant frequency (the primary analysis of this study) was not performed. Independently of this prevalence-oriented calculation, a target of 100 patients per group was pre-specified in the study design to ensure adequate representation of both arms of the primary between-group comparison. Group 1 (discharged ≤24 h after ERCP) and Group 2 (discharged >24 h after ERCP) are defined by an outcome observed only after ERCP. Patients presenting for ERCP during the study period (July 2016–February 2019) were therefore assessed and enrolled consecutively. Once a given group’s quota of 100 was reached, subsequently eligible patients whose outcome would have placed them in that already-filled group were not included in the analytic cohort. Enrollment continued for the other group until it, too, reached 100. A total of 200 patients were included in the study. Group size was therefore fixed by this pre-specified quota rather than by the natural relative frequency of each outcome in the source population. As a result, this cohort cannot be used to estimate the overall prevalence of NOD2 variants, the distribution of length of stay, or the frequency of prolonged hospitalization at alternative discharge-time cutoffs in the source population of ERCP patients after LDLT (see Section 4). The odds ratio for the pre-specified 24 h comparison is less directly affected by this design, though residual dependence on within-group selection cannot be excluded.

### 2.2. Patients

Liver transplant patients with live donors and duct-to duct anastomoses who underwent endoscopic retrograde cholangiopancreatography (ERCP) in the Gastroenterology Department of Turgut Ozal Medical Center, Malatya, Turkey between July 2016 and February 2019 were included in the current study. Patients > 18 years of age were included in the study. Ethics Committee approval was received for our study (protocol number: 2016/145). All patients usually used a tacrolimus-based immunosuppressive regime.

Before ERCP, every patient was evaluated by a multidisciplinary team including transplant surgeons, radiologists, and gastroenterologists. This evaluation incorporated clinical data and imaging, including MRI or catheter cholangiography. Biliary complications were suspected on the basis of clinical symptoms (jaundice, itching, and cholangitis), deranged liver function tests (bilirubin, transaminases, and/or gamma-glutamyl transferase levels), or radiologic imaging. Biliary stricture was suspected if there were symptoms of cholangitis or elevation of liver function tests. Magnetic resonance cholangiopancreatography (MRCP), computerized tomography (CT), or transhepatic biliary catheter cholangiography was performed to diagnose biliary strictures. If there were findings of biliary stricture, ERCP was preferred as the first choice in patients with duct-to-duct anastomosis. All ERCP procedures were performed by a single experienced gastroenterologist using Olympus duodenoscopes (TJF 160, Olympus Optical Co., Ltd., Tokyo, Japan). Informed written consent for the ERCP procedure was obtained directly from each patient. In a small number of patients with whom consistent communication could not be established because of a language barrier, consent was instead obtained from their legal guardian, as provided for under the approved study protocol (2016/145). An intravenous prophylactic antibiotic (4.5 g piperacillin-tazobactam) was administered before all procedures. Antibiotic treatment was continued in patients whose stricture could not be passed until biliary drainage was achieved. We performed endoscopic sphincterotomy routinely in every patient during ERCP. A 0.025 or 0.035-in. guidewire (Jagwire, Boston Scientific, Natick, MA, USA) was inserted via catheter into the intrahepatic bile ducts if biliary stricture is detected on contrast cholangiography. Then, we dilated it using a Bougie (7 and 10 French, Wilson-Cook Medical GI Endoscopy, Winston-Salem, NC, USA) and/or a balloon catheter (4, 6, or 8 mm, Hurricane RX; Boston Scientific, Natick, MA, USA). Then, Amsterdam-type biliary stents (7 and 10 French, 9–16 cm long, Boston Scientific) were placed across the stricture or anastomosis.

Patients who had all these procedures were successful and did not have any complaints after the procedure were usually discharged within the first 24 h. Transplant patients whose biliary stricture could not be passed continued to be hospitalized for antibiotic treatment and the second session of ERCP. Because discharge timing was therefore governed in part by this institutional policy linking stricture-passage outcome to disposition, Group 2 membership is partially determined by stricture-passage failure itself; the comparison of stricture-passage failure between groups (see Section 3) is consequently a direct reflection of this policy rather than an independent clinical association, and is reported for descriptive completeness rather than as a hypothesis-driven finding. We took these parameters into consideration while prospectively dividing patients into two groups. Specifically, the groups were as follows: Group 1 (*n* = 100), patients discharged during the first 24 h after ERCP; and Group 2 (*n* = 100), patients discharged after the first 24 h following ERCP. Patients were enrolled prospectively and assigned to Group 1 or Group 2 according to their observed discharge timing; enrollment continued until each group reached its target of 100 patients, rather than following a strict, uninterrupted consecutive-series design (see Section 4). Informed consent for study participation was obtained directly from all patients. In a small number of patients with whom consistent communication could not be established because of a language barrier, consent was instead obtained from their legal guardian, as provided for under the approved study protocol (2016/145). The patient flow is shown in Figure 1.

### 2.3. Sample Collection

The blood samples of the patients included in our study were obtained on the day of the ERCP procedure. Venous blood samples were collected in bottles containing 9 mL of EDTA and used in the analysis of NOD2 risk variants. We also obtained blood and serum samples, with EDTA used as an anticoagulant, for standard hematologic, biochemical, and coagulation testing. Samples were subjected to the relevant biochemical analyses or stored at −70 °C until used. White blood cell (WBC), C-reactive protein (CRP), total bilirubin (T.Bil), alkaline phosphatase (ALP), gamma-glutamyl transferase (GGT), blood urea nitrogen (BUN), creatinine, prothrombin time (PT), the international normalized ratio (INR), amylase, and lipase values were determined by routine laboratory testing.

### 2.4. Molecular Methods

#### Detection of NOD2 Gene Mutations

NOD2 genotyping was performed in batches from frozen EDTA blood samples after each patient’s ERCP outcome and discharge disposition had already been determined; treating clinicians and patients were therefore unaware of NOD2 genotype results at the time discharge decisions were made, precluding genotype-informed clinical decision-making as a source of bias. A real-time PCR method developed by Appenrodt et al. [15] was used to analyze the most frequently detected NOD2 variants (p.R702W, p.G908R, and c.3020insC) in the samples. In addition, the 1007fs variant reported by Bruns et al. [16] was included for analysis. Nucleotide-level (c.3020insC) and protein-level frameshift (1007fs) nomenclature both refer to the same NOD2 variant (rs2066847, SNP13). This variant was independently assayed using the two protocols above, which target overlapping regions of exon 11 with different probe chemistries. The Appenrodt-protocol probe did not detect any carriers of this variant in this cohort, whereas the Bruns-protocol probe detected 17 carriers, each individually confirmed by Sanger sequencing. Because the two assays target the same underlying variant, only the sequencing-confirmed Bruns-protocol result (1007fs) is reported as the definitive carrier count for this locus; the discordant Appenrodt-protocol result is not reported separately (see Section 4). The primers and probes used to detect NOD2 gene mutations are presented in Table 1.

The QuantiTect Probe RT-PCR kit (Lot no. 154027285; Qiagen, Hilden, Germany) was used for real-time PCR analysis. Each reaction contained the appropriate primer (0.4 μM) and probe (0.2 μM). Amplification was performed in a Rotor-Gene Q Real device (Qiagen) under the following conditions: initial denaturation at 95 °C for 15 min followed by denaturation at 94 °C for 15 s for 40 cycles; and annealing (fluorescent measurements) at 60 °C for 1 min. Mutations in the samples positive for NOD2 variant(s) were confirmed with DNA sequencing with an ABI PRISM 310 device (Applied Biosystems, Foster City, CA, USA).

### 2.5. Sequencing

Primers targeting the mutated regions were generated. Mutations were confirmed by sequencing the amplified DNA regions via PCR using a BigDye Terminator 3.1 kit (Applied Biosystems) and the ABI PRISM 310 automatic base sequencing device (Applied Biosystems).

### 2.6. Statistical Analysis

Data are shown as the median (min–max) and number (percentage). Compatibility with a normal distribution was determined with the Kolmogorov–Smirnov test. For statistical analyses, the Mann–Whitney U test, Pearson chi-square test, Yates’ corrected chi-square test, and Fisher exact chi-square tests were used as indicated. The relationship between qualitative variables is reported as the Phi coefficient. In all evaluations, a *p* < 0.05 value was considered statistically significant. IBM SPSS Statistics 25.0 (IBM Corp., Armonk, NY, USA) was used in the analysis. Firth’s penalized logistic regression, negative binomial regression, and log-linear regression with heteroscedasticity-robust (HC3) standard errors were performed in Python (Python Software Foundation, Wilmington, DE, USA; https://www.python.org, accessed on 23 September 2026) using open-source scientific libraries, including NumPy, SciPy, and statsmodels (https://www.statsmodels.org, accessed on 23 September 2026). To account for multiple comparisons across the laboratory parameters (Table 2) and NOD2 variants (Table 3), the Benjamini–Hochberg false discovery rate (FDR) procedure was additionally applied within each family of tests, and FDR-adjusted *p* values are reported alongside nominal *p* values. For the multivariable model restricted to patients with successful stricture passage, age, CRP, and IL-6 were selected a priori as covariates because CRP and IL-6 reflect the systemic host inflammatory response through which NOD2 is hypothesized to act—IL-6 stimulates hepatocytes to synthesize CRP as part of the acute-phase response [17]—whereas the other laboratory parameters that differed between groups (e.g., bilirubin, ALP, GGT, amylase, and lipase) primarily reflect local biliary or pancreatic injury and were considered potential mediators, rather than independent confounders, of the stricture-related pathway; adjusting for such mediators could have inappropriately attenuated the estimated effect of NOD2 variants. Age was included as a standard demographic covariate. The two genetically distinct NOD2 variants identified in our cohort, p.R702W and 1007fs, were combined into a single binary predictor (variant present vs. absent) for the multivariable models, rather than modeled separately, because p.R702W alone produced quasi-complete separation in the stricture-passed subgroup (all 7 carriers in this subgroup were in the prolonged hospitalization group), which precluded stable separate effect estimation even with Firth’s correction. This combined-predictor approach should be interpreted as reflecting the presence of any high-risk NOD2 variant rather than a variant-specific effect; unadjusted, variant-specific frequencies and odds ratios are reported separately in Table 3.

## 3. Results

Time to ERCP after transplantation, gender, and age of the individuals included in our study are shown in Table 4. There was no significant difference for these parameters between the groups. The median length of hospital stay in the group hospitalized for a long time was 4.5 days (range, 2–30 days). The frequency of patients whose biliary strictures in which the endoscopic intervention could not be passed during ERCP is also shown in Table 4. The number of patients in the group discharged after the first 24 h following ERCP in whom endoscopic intervention could not pass the strictures was significantly higher than the other group (34% vs. 0%, *p* < 0.0001). This difference is an expected, direct consequence of the institutional discharge policy described in Section 2.2, under which stricture-passage failure itself prompted continued hospitalization; it is reported descriptively rather than as an independent finding.

Comparisons of the WBC, CRP, T.Bil, ALP, GGT, BUN, creatinine, INR, amylase, and lipase levels of the individuals included in our study are shown in Table 2. A significant difference was observed between the groups for all parameters, with the exception of creatinine and INR.

The frequency of NOD2 mutations by groups is shown in Table 3. No p.G908R mutations were detected in any patient included in the study. In the current study, p.R702W mutation was not detected in any patients who were discharged during the first 24 h after ERCP; however, this mutation was detected in ten patients in the group discharged after the first 24 h following ERCP. The frequency of the p.R702W mutation between the two groups was statistically different (*p* = 0.002). The 1007fs mutation was detected in 3 patients in the group discharged during the first 24 h after ERCP; however, this mutation was detected in 14 patients in the group discharged after the first 24 h following ERCP. The frequency of the 1007fs mutation between the two groups was statistically different (*p* = 0.011). Both associations remained statistically significant after Benjamini–Hochberg FDR correction for multiple comparisons across the three NOD2 variants (adjusted *p* = 0.005 and *p* = 0.017, respectively; Table 3). The corresponding odds ratios were 23.32 (95% CI: 1.35–403.67; Haldane–Anscombe corrected owing to a zero cell) for p.R702W and 5.26 (95% CI: 1.46–18.94; computed without continuity correction, as no cell was zero) for 1007fs (Table 3).

Analysis of the factors affecting the length of hospital stay revealed a moderate relationship between long hospitalization (Group 2; patients discharged after the first 24 h following ERCP) and failure of the endoscopic intervention to pass the stricture during ERCP (Phi value: 0.453; Table 5). The relationship between the p.R702W variant and long hospitalization was weaker compared to the failure of the endoscopic intervention to pass the stricture during ERCP (Phi value: 0.229). The weakest relationship among the factors affecting long hospitalization involved the 1007fs mutation (Phi value: 0.197).

Restricting the multivariable model to patients in whom the stricture was successfully passed (as in the analysis above) could introduce selection (collider) bias, because NOD2 variants may themselves influence the severity of the underlying stricture and, consequently, the likelihood of successful endoscopic passage. To address this, we additionally fit a multivariable model including all 200 patients, using Firth’s penalized (bias-reduced) logistic regression to handle the quasi-complete separation caused by the near-perfect association between stricture-passage failure and prolonged hospitalization. In this full-cohort model, NOD2 variant carriage remained strongly associated with prolonged hospitalization after adjustment for the measured covariates (OR: 14.55, 95% CI: 3.06–69.04, *p* < 0.001; Table 6), including stricture-passage failure, age, and log-transformed CRP and IL-6.

As a sensitivity analysis, we additionally restricted the model to the 166 patients in whom the stricture was successfully passed endoscopically (Table 7), adjusting for age, CRP, and IL-6. NOD2 variant carriage remained associated with prolonged hospitalization, after adjustment for the measured covariates, in this subgroup (odds ratio [OR]: 11.02, 95% confidence interval [CI]: 2.24–54.08, *p* = 0.003), consistent with the full-cohort model (Table 6) and further supporting that this association is not attributable solely to failure of stricture passage.

Because dichotomizing hospital stay at 24 h discards information about the actual duration of hospitalization, we additionally modeled the raw number of hospitalization days as a continuous outcome, using both a log-linear regression (with heteroscedasticity-robust standard errors) and a negative binomial regression, adjusting for stricture-passage failure, age, and log-transformed CRP and IL-6 (Table 8). In these continuous-outcome models, NOD2 variant carriage showed a positive but statistically non-significant association with length of stay (fold-change: 1.27, 95% CI: 0.89–1.81, *p* = 0.182 in the log-linear model; incidence rate ratio: 1.43, 95% CI: 0.88–2.31, *p* = 0.146 in the negative binomial model), whereas stricture-passage failure and CRP remained strong, significant predictors in both models. This contrasts with the strong, significant association observed when hospitalization was dichotomized at 24 h (Table 6 and Table 7), and should be reported transparently rather than omitted. We consider two non-mutually exclusive explanations. First, the effect of NOD2 variants may be more closely tied to whether a specific clinical threshold (e.g., readiness for discharge at 24 h) is crossed than to a proportional, dose–response relationship with total days hospitalized. Second, statistical power for the continuous-outcome models may have been limited by the small number of NOD2 variant carriers (*n* = 27) relative to the number of covariates. Consequently, the association observed at the 24 h discharge threshold should not be assumed to reflect a proportional, continuous relationship with length of stay; whether it holds across a range of clinically relevant thresholds, or is specific to the 24 h cutoff used to define our primary outcome, is examined further below using alternative discharge-time thresholds (Table 9).

To further evaluate the robustness of the NOD2–hospitalization association to the specific dichotomization used, we repeated the full-cohort Firth model with prolonged hospitalization redefined at >48 h and >72 h thresholds instead of the original >24 h cutoff (Table 9). The magnitude of the NOD2 association weakened progressively and lost statistical significance at both alternative cutoffs (OR: 2.89, 95% CI: 0.90–9.27, *p* = 0.075 at >48 h; OR: 1.99, 95% CI: 0.64–6.16, *p* = 0.235 at >72 h), whereas stricture-passage failure and CRP remained significant at every threshold tested. Taken together with the continuous-outcome analysis (Table 8), these findings indicate that the association between NOD2 variants and prolonged hospitalization is strongest, and reaches statistical significance, specifically around the 24 h discharge threshold used to define our primary outcome; it should therefore be regarded as a cutoff-dependent finding that warrants replication with prespecified, clinically motivated thresholds in independent cohorts, rather than as evidence of a general, dose-dependent effect of NOD2 variants on hospitalization duration.

## 4. Discussion

In our study, two NOD2 variants (p.R702W and 1007fs) were significantly more frequent in the group discharged after the first 24 h following ERCP. Endoscopic treatment failure in patients undergoing ERCP due to biliary strictures after LDLT is an important factor leading to long hospitalization. Endoscopic treatment failure is usually due to an inability of the endoscopic intervention to pass a stricture. In our study, the number of patients whose strictures could not be passed with endoscopic intervention in Group 2 was significantly higher than the other group (34% vs. 0%, *p* < 0.0001). This association reflects the discharge policy itself—patients whose stricture could not be passed were, by protocol, kept hospitalized for antibiotic treatment and a second ERCP—rather than an independent clinical relationship; it should therefore be interpreted as confirming adherence to the discharge criterion rather than as a novel finding linking stricture-passage failure to length of stay. In contrast, the NOD2–hospitalization association at the 24 h threshold reflects an association with actual length of stay after adjustment for the measured covariates, rather than a definitional consequence of the discharge policy, as assessed by the full-cohort model (Table 6) and the stricture-passed sensitivity analysis (Table 7). This association is itself cutoff-dependent: as detailed below, it was not observed when length of stay was analyzed continuously (Table 8) or at alternative discharge-time thresholds (Table 9). In the sub-analysis, an association was demonstrated between the presence of a NOD2 variant and the length of hospital stay. This relationship was not as strong as the relationship between the length of hospitalization and failure of the endoscopic intervention to pass a stricture. When the NOD2 variants were compared, there was an apparent relationship between the p.R702W variant and a prolonged hospital stay. Because restricting this analysis to patients with successful stricture passage could itself introduce selection (collider) bias—as NOD2 may influence stricture severity and thus passage success —we additionally fit a multivariable Firth (bias-reduced) logistic regression including all 200 patients, adjusting for stricture-passage failure, age, CRP, and IL-6. In this full-cohort model, NOD2 variant carriage remained strongly associated with prolonged hospitalization after adjustment for the measured covariates (OR: 14.55, 95% CI: 3.06–69.04, *p* < 0.001; Table 6). This finding was corroborated by a sensitivity analysis restricted to patients with successful stricture passage (OR: 11.02, 95% CI: 2.24–54.08, *p* = 0.003; Table 7), indicating that the association between NOD2 variants and prolonged hospitalization is not solely attributable to failure of stricture passage.

Liver transplantation is a life-saving treatment modality in the treatment of acute and chronic liver failure. Currently, despite all technical and scientific advances, biliary complications still have a significant effect on patient outcomes after liver transplantation. These complications are a more severe problem, especially after LDLT. The underlying basis for biliary strictures includes ischemia or fibrosis following a suboptimal surgical technique or a bile leak in the post-operative period. In the early post-operative period, strictures usually depend on technical factors; however, a late onset of anastomotic strictures most likely indicate fibrotic healing arising from ischemia at the end of the donor or recipient bile duct [18]. In patients with strictures after LDLT, the main reason for prolonged hospitalization after ERCP is that the stricture cannot be passed endoscopically. There are many studies focusing on the results of endoscopy treatment [19,20] in patients with biliary complications after LT; however, there is no study on the effects of host factors (especially the host’s immune system) on the length of hospital stay in this patient group. NOD2 is a component of the innate immune system and an intracellular pattern recognition receptor that senses bacterial peptidoglycan-conserved motifs in the cytosol and stimulates the host immune response [21]. It has been shown that defective NOD2 sensing increases intestinal translocation and bacterial invasion in the liver, thus leading to inflammation [22]. Bacteria may penetrate the damaged mucosal layer during acute inflammatory episodes, enter the liver, and stimulate the release of chemokines/cytokines by Kupffer cells and macrophages, leading to cholangitis and the resulting wound healing process with concentric periductal fibrosis [23]. Jüngst et al. [14] determined whether or not NOD2 gene variants create a risk for secondary sclerosing cholangitis in critically ill patients, and estimated that NOD2 variant carriers had at least a 2-fold increased risk for secondary sclerosing cholangitis in critically ill patients. Similarly, in our previous studies [12,23], we reported that there might be a relationship between NOD2 variants and bacterial translocation in patients with severe pancreatitis and cirrhosis. In the current study, we investigated the effect of three NOD2 variants, as host factors, on the duration of hospitalization in patients with biliary complications after LDLT. We found that two NOD2 variants (p.R702W and 1007fs) were significantly more frequent in the group discharged after the first 24 h following ERCP. These findings suggest that there may be a relationship between bacterial translocation, which develops based on NOD2 variants as a host factor, and ongoing cholangitis and fibrosis in bile ducts. It should be emphasized that this mechanistic pathway is a hypothesis derived from prior experimental literature; bacterial translocation, cholangitis severity, and periductal fibrosis were not directly measured in the present study, and our findings should not be interpreted as evidence for this specific mechanism. Moreover, because all patients in this cohort already had biliary stricture and no comparison group without biliary complications was studied, these data cannot establish whether NOD2 variants predispose to the development of biliary complications or periductal fibrosis; they speak only to the association between NOD2 variant carriage and postoperative length of stay among patients who had already developed stricture.

A further consideration is that this study, like the recipient-genotype design used by Janse et al. [13], assessed only the recipient’s NOD2 genotype. Because LDLT involves duct-to-duct biliary anastomosis and the biliary epithelium at the anastomotic site is partly donor-derived, donor NOD2 genotype could also plausibly influence local biliary inflammation, healing, and stricture formation, independently of the recipient’s systemic immune genotype assessed here. Donor genotyping was not available in this cohort, and we therefore cannot exclude the contribution of donor NOD2 status to the observed associations; this remains an important direction for future studies of NOD2 in the LDLT setting.

There are very few studies investigating how NOD2 variants affect liver transplant patients [13,24,25,26]. Although a study reported that 50 common genetic variations, including NOD2, in innate immunity receptors do not influence susceptibility to bacterial/fungal infections and acute rejection after liver transplantation (LT) [24], Janse et al. [13] reported that the mutated NOD2 R702W genotype in the recipient is associated with an increased risk of bacterial infections after LT. In another study, it was shown that NOD2 is involved in promoting or attenuating hepatic ischemia–reperfusion injury in liver transplant patients [25]. Furthermore, it was reported that diagnostic screening for pattern recognition receptors, such as NOD2, might prove useful for prediction and/or therapeutic intervention in liver transplant patients before transplantation [25]. Saner et al. [26] investigated the effect of NOD2 variants on survival in liver transplant patients and found that non-alcoholic patients with wild-type NOD2 genes had a 1-year survival rate of 75.4%, whereas patients with polymorphisms had a survival rate of 49.7%. Because of this statistically significant difference, Saner et al. [26] suggested that patients with the NOD2 gene variant should have closer follow-up to satisfy the standard of care. In all of these studies, the LT type was cadaveric; however, all of the patients we included in our study were LDLT. Thus far there are no studies in the literature investigating the relationship of NOD2 in LDLT patients. In our study, we did not investigate the frequency of bacterial infections and survival, but found that NOD2 variants, especially p.R702W, were more common in the group who were hospitalized for a long time. In the light of all data, our findings raise a hypothesis-generating possibility. Among patients who develop biliary stricture after LDLT, NOD2 genotyping could eventually inform risk stratification for discharge beyond the 24 h threshold following ERCP—the cutoff-dependent outcome demonstrated in this study. This possibility does not extend to continuous length of stay, which was not significantly associated with NOD2 variant carriage (Table 8). This is not a validated clinical risk-stratification tool. Because this cohort did not include a comparison group without biliary stricture, our data do not support using NOD2 genotyping to predict the development of biliary complications themselves. Given the single-center design, modest sample size, and small number of variant carriers in our cohort, even this narrower application would require validation in larger, multicenter studies before any diagnostic or prophylactic use could be considered.

The effects of NOD2 variants were also investigated in transplant patients other than LT. One study reported no relationship between NOD2 variants and adverse outcomes in patients with bone marrow transplantation [27]; however, studies have reported that NOD2 variants have an effect on the occurrence of graft-versus-host disease after bone marrow transplantation [28,29]. In one study, NOD2 variants were shown to be associated with higher rates of death after kidney transplantation [30]. Although one study reported that NOD2 variants did not affect the results in small bowel transplant patients [31], there are also studies that have reported that NOD2 variants decrease graft and patient survival in these patients [32,33]. In one of these studies, Ningappa et al. [33] reported that an association existed between a NOD2 variant (R702W) with early intestinal transplant rejection and septic death. All of these studies suggest that NOD2 variants play an important role in adverse outcomes in transplant patients, as we have obtained in the current study.

Our study has some limitations. First, while dividing patients into groups, we only considered the length of stay in the hospital after ERCP. We did not consider how many times ERCP was applied to patients for biliary complications. However, the number of ERCP sessions can significantly affect the duration of hospitalization of patients after ERCP. Second, it was difficult to determine the most appropriate method to investigate the effect of NOD2 variants on hospital stay in LDLT patients with biliary complications, because biliary complications are the Achilles’ heel of liver transplantation. Biliary complications are one of the most important complications affecting long-term results in transplant patients. The low endoscopic success rate for biliary complications in LDLT patients can be attributed to multiple, small-caliber anastomoses, peripheral locations, and twisted structures, which probably result from anastomotic fibrosis and hypertrophy of the transplanted liver [34]. Moreover, liver transplant patients use immunosuppressive therapy throughout their life. All of this explains why it is difficult to determine the most appropriate method for investigating the impact of NOD2 variants on hospital stay in LDLT patients with biliary complications. Third, the sample size calculation was performed to ensure adequate precision for estimating the overall prevalence of NOD2 variants in this population, rather than as an a priori power calculation for the between-group comparison that constitutes our primary analysis; the study may therefore have been underpowered to detect between-group differences smaller than those observed. Fourth, only carrier counts were recorded for each NOD2 variant; genotype-level detail (zygosity, allele frequencies), call rates, and duplicate- or sequencing-based genotyping concordance were not systematically assessed, and Hardy–Weinberg equilibrium could therefore not be evaluated. In addition, all patients in this cohort had already developed biliary stricture requiring ERCP; because no comparison group without biliary stricture was included, this study cannot address whether NOD2 variants predispose to the development of biliary complications or periductal fibrosis, and our conclusions are restricted to associations with postoperative course (length of hospital stay) among patients who had already developed stricture. Fifth, because the primary multivariable model (Table 6) relied on Firth’s penalized likelihood to address quasi-complete separation and included a limited number of events for some strata (e.g., only 10 patients carried p.R702W), the resulting confidence intervals—while appropriately wide—should be interpreted with caution, and replication in larger, multicenter cohorts is warranted. Sixth, the c.3020insC and 1007fs nomenclatures refer to the same underlying NOD2 variant (rs2066847), which was assayed here by two independent real-time PCR protocols targeting overlapping regions; the Appenrodt-protocol assay for this variant yielded discordant (uniformly negative) results relative to the sequencing-confirmed Bruns-protocol assay, likely reflecting differences in probe performance between the two protocols. Only the sequencing-confirmed result (1007fs) is reported in Table 4. This discordance underscores the importance of orthogonal confirmation (e.g., sequencing) when assaying rare variants with multiple historical nomenclatures. Seventh, several clinical variables that could plausibly affect both ERCP outcome and length of stay were not recorded and could therefore not be adjusted for, including stricture location and length, anastomotic anatomy, the number of prior ERCP sessions, stent history, active cholangitis, bacteremia or bile culture results, hepatic artery complications, bile leak, donor and graft characteristics, and the intensity of immunosuppression (including tacrolimus levels); the associations reported here should therefore be interpreted as adjusted for the covariates measured rather than as fully independent of all possible confounding. Eighth, immunosuppression was characterized only as “usually tacrolimus-based”; more granular data on immunosuppressive regimen, dosing, or trough levels were not available for this cohort. Ninth, Group 1 and Group 2 (defined by discharge timing) were each enrolled to a pre-specified quota of 100 patients. Eligible patients were assessed consecutively as they presented for ERCP throughout the study period, but once a given group’s quota was reached, subsequently eligible patients whose outcome would have placed them in that group were not included in the analytic cohort. Because of this quota-based design, estimates of the overall prevalence of NOD2 variants, the distribution of length of stay (Table 8), and the frequency of prolonged hospitalization at alternative discharge-time cutoffs (Table 9) may reflect the sampling process rather than the source population of ERCP patients after LDLT, and should not be generalized beyond this cohort. The odds ratio for the pre-specified 24 h comparison is less directly affected, though residual dependence on within-group selection cannot be excluded. The exact number of patients assessed and excluded for this reason, and the enrollment date range for each group, could not be fully reconstructed retrospectively and are therefore not reported in Figure 1. Findings should accordingly be regarded as exploratory and require confirmation in independent, consecutively enrolled cohorts with complete accrual records. Tenth, positive real-time PCR calls for p.R702W and 1007fs were confirmed by Sanger sequencing (see Section 2.4 and Section 2.5). Negative calls, including the complete absence of p.G908R across all 200 patients, were not independently reconfirmed by sequencing or checked against defined positive-control call criteria beyond the assay’s internal fluorescence threshold. We therefore cannot fully exclude assay-related false negatives for these calls.

While there are many NOD2 variants identified thus far, three variants (p.G908R, p.R702W, and 1007fs) were investigated more intensively in studies related to NOD2 variants; we therefore preferred these variants in our study. We compared NOD2 variants and found a more significant relationship between the p.R702W variant and a long hospital stay. Janse et al. [13] reported that the NOD2 R702W variant in the recipient is associated with an increased risk of bacterial infections after LT. The R702W variant has been shown to be associated with rejection and septic death after small bowel transplantation [33]. Additionally, Jüngst et al. [14] reported an association between the R702W variant and secondary sclerosing cholangitis in critically ill patients. All this suggests that the p.R702W variant is more prominent than other NOD2 variants. Screening for this variant may eventually be useful specifically in patients who, like ours, already have a biliary stricture and are undergoing ERCP after LDLT, pending validation in larger, multicenter cohorts; our findings do not support broader screening of liver transplant patients in general.

In conclusion, our findings suggest that there may be a relationship between NOD2 variants, especially the p.R702W variant, and length of hospital stay in patients undergoing ERCP after LDLT when defined by the 24 h discharge threshold used here. Because this association was not confirmed in the continuous-outcome or alternative-threshold analyses (Table 8 and Table 9), it should be regarded as cutoff-dependent and requires replication with prespecified, clinically motivated thresholds in larger, multicenter cohorts before broader conclusions can be drawn.

## Figures and Tables

**Figure 1:**
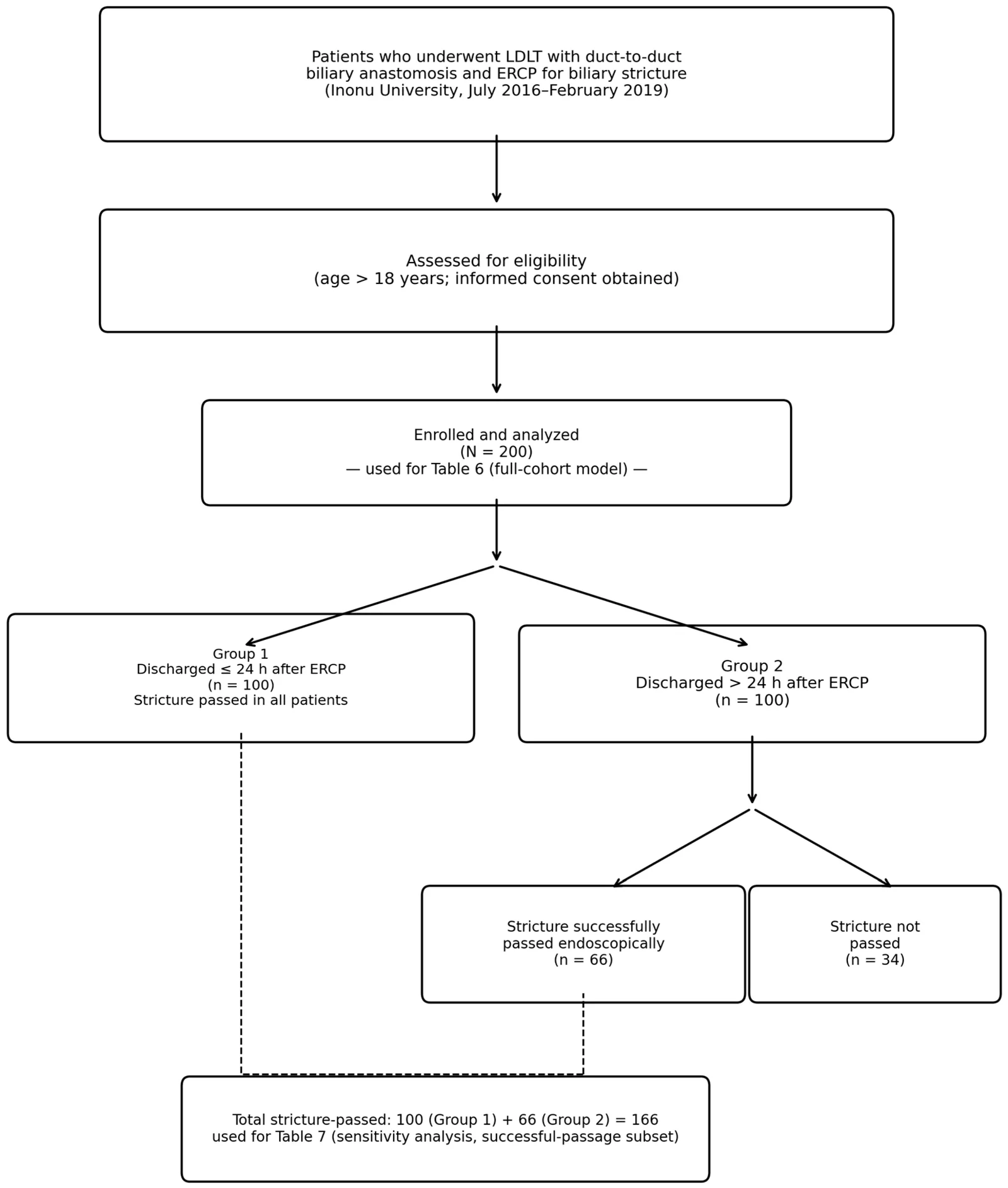
Patient flow diagram. Pre-enrollment screening and exclusion counts, and group-specific enrollment date ranges, could not be fully reconstructed retrospectively and are therefore not depicted (see Section 4).

**Table 1:** Primers and probes used for NOD2 genotyping.

NOD2 Variant	Primer/Probe	Sequence (5′→3′)
p.R702W	MGB_F	CTGAGTGCCAGACATCTGAGAAG
	MGB_R	GCTGCGGGCCAGACA
	VIC probe	CCTGCTCTGGCGCC
	FAM probe	CTGCTCCGGCGCC
p.G908R	MGB_F	TGATCACCCAAGGCTTCAGC
	MGB_R	GAACACATATCAGGTACTCACTGACAC
	VIC probe	ACTCTGTTGCGCCAGA
	FAM probe	CTGTTGCCCCAGAAT
c.3020insC	MGB_F	CCAGGTTGTCCAATAACTGCATC
	MGB_R	CCTTACCAGACTTCCAGGATGGT
	VIC probe	TGCAGGCCCCTTG
	FAM probe	CTGCAGGCCCTTG
1007fs	Forward primer	GTCCAATAACTGCATCACCTACCTAG
	Reverse primer	CTTACCAGACTTCCAGGATGGTGT
	Probe 1 (FAM)	CCCTCCTGCAGGCCCTTGAAAT
	Probe 2 (VIC)	CCTCCTGCAGGCCCCTTGAAA

Note: The c.3020insC and 1007fs assays listed above target the same NOD2 variant (rs2066847, SNP13) via overlapping primer/probe sets; see Section Detection of NOD2 Gene Mutations for the resolution of assay discordance between these two protocols.

**Table 2:** Laboratory parameters by group.

Parameter	Group 1	Group 2	*p*	Adjusted *p* (BH-FDR)
WBC (10^9^/L)	5.90 (3.20–10.80)	7.00 (1.90–40.10)	0.003	0.004
CRP (mg/dL)	0.30 (0.30–8.20)	3.35 (0.30–30.70)	<0.001	<0.001
Total bilirubin (mg/dL)	0.90 (0.19–2.80)	2.50 (0.25–42.00)	<0.001	<0.001
ALP (U/L)	131.50 (48.00–827.00)	274.00 (11.00–2234.00)	<0.001	<0.001
GGT (U/L)	52.50 (8.00–427.00)	240.00 (15.00–3627.00)	<0.001	<0.001
BUN (mg/dL)	9.95 (1.60–38.50)	12.30 (5.00–42.00)	0.022	0.027
Creatinine (mg/dL)	0.80 (0.54–2.80)	0.80 (0.55–77.00)	NS	NS (0.376)
INR	1.06 (0.78–1.80)	1.10 (0.80–2.07)	NS	NS (0.130)
Amylase (U/L)	36.00 (10.00–122.00)	47.50 (5.00–1967.00)	<0.001	<0.001
Lipase (U/L)	16.00 (0.14–64.00)	23.00 (5.00–4428.00)	0.002	0.003
IL-6 (pg/mL)	0.00 (0.00–92.00)	5.00 (0.00–13,922.00)	<0.001	0.001

Values are median (min–max). *p* values were adjusted for multiple comparisons across the 11 laboratory parameters using the Benjamini–Hochberg false discovery rate (FDR) procedure.

**Table 3:** Frequency of NOD2 mutations by group.

NOD2 Mutation	Group 1	Group 2	*p*	Adjusted *p* (BH-FDR)	OR (95% CI)
p.G908R	0 (0%)	0 (0%)	NS	NS	NA
p.R702W	0 (0%)	10 (10%)	0.002	0.005	23.32 (1.35–403.67) *
1007fs	3 (3%)	14 (14%)	0.011	0.017	5.26 (1.46–18.94)

OR: odds ratio; CI: confidence interval; NA: not applicable (no events in either group). * Because no p.R702W-positive patients were observed in Group 1 (zero cell), the Haldane–Anscombe continuity correction (+0.5 to each cell) was applied to estimate the OR and 95% CI; the resulting wide interval reflects the sparse data for this comparison and should be interpreted with caution. The 1007fs OR and 95% CI were computed from the observed cell counts without continuity correction, as no cell was zero.

**Table 4:** Demographic characteristics and ERCP stricture-passage outcome by group.

	Group 1 (*n* = 100)	Group 2 (*n* = 100)	*p*
Gender (F/M)	74/26	68/32	NS
Age (years)	49.50 (18.00–69.00)	53.00 (20.00–76.00)	NS
Time from transplantation to ERCP (months)	29.00 (2.00–239.00)	20.00 (1.00–137.00)	NS
Stricture not passed endoscopically, *n* (%)	0 (0%)	34 (34%)	<0.0001

Values are median (min–max) for continuous variables. F: female; M: male; NS: *p* > 0.05.

**Table 5:** Relationship (Phi coefficient) between ERCP findings, NOD2 variants, and length of hospitalization.

Factor	Group 1 (*n*)	Group 2 (*n*)	Phi Coefficient
Stricture not passed	0	34	0.453
p.R702W	0	10	0.229
1007fs	3	14	0.197

Interpretation of the Phi coefficient: 0.00–0.19, no or negligible relationship; 0.20–0.39, weak relationship; 0.40–0.69, moderate relationship; 0.70–0.89, strong relationship; 0.90–1.00, very strong relationship.

**Table 6:** Multivariable Firth (bias-reduced) logistic regression for prolonged hospitalization, full cohort (*n* = 200).

Variable	OR	95% CI	*p*
NOD2 variant (p.R702W or 1007fs)	14.55	3.06–69.04	<0.001
Stricture not passed endoscopically	22.65	0.55–926.80	0.099
log (CRP+1)	59.89	15.41–232.71	<0.001
Age	0.996	0.96–1.04	NS
log(IL-6+1)	1.23	0.94–1.60	NS

Firth’s penalized (bias-reduced) logistic regression was used because stricture-passage failure was quasi-completely separated from the outcome (all 34 patients with unpassed strictures had prolonged hospitalization), and standard maximum-likelihood estimation did not converge. All 200 patients were included, avoiding the need to exclude patients with failed stricture passage—an exclusion that could otherwise introduce selection (collider) bias, since NOD2 may itself influence stricture severity and thus passage success. CRP and IL-6 were log-transformed (log[x + 1]) owing to marked right-skew. OR: odds ratio; CI: confidence interval.

**Table 7:** Sensitivity analysis: multivariable logistic regression for prolonged hospitalization restricted to patients with successful stricture passage (*n* = 166).

Variable	OR	95% CI	*p*
NOD2 variant (p.R702W or 1007fs)	11.02	2.24–54.08	0.003
CRP	3.94	2.24–6.94	<0.001
Age	1.00	0.96–1.04	NS
IL-6	1.02	1.00–1.04	NS (0.10)

Model adjusted for age, CRP, and IL-6; restricted to the subset of patients in whom the stricture was passed endoscopically, as a sensitivity analysis complementing the full-cohort model in Table 6. OR: odds ratio; CI: confidence interval.

**Table 8:** Multivariable models for length of hospital stay as a continuous outcome (*n* = 200).

Model	Variable	Effect (95% CI)	*p*
Log-linear regression (fold-change in LOS)	NOD2 variant (any)	1.27 (0.89–1.81)	0.182
	Stricture not passed	2.08 (1.44–3.01)	<0.001
	log(CRP+1)	2.04 (1.71–2.44)	<0.001
Negative binomial (incidence rate ratio)	NOD2 variant (any)	1.43 (0.88–2.31)	0.146
	Stricture not passed	2.02 (1.27–3.20)	0.003
	log(CRP+1)	2.15 (1.71–2.70)	<0.001

Both models adjusted for stricture-passage failure, age, and log-transformed CRP and IL-6. LOS: length of stay. Log-linear model: outcome was log-transformed LOS; effect is the multiplicative fold-change in LOS per predictor. Negative binomial model: effect is the incidence rate ratio. Standard errors for the log-linear model are heteroscedasticity-robust (HC3).

**Table 9:** Sensitivity analysis: association between NOD2 variant carriage and prolonged hospitalization across alternative discharge-time cutoffs (Firth full-cohort models, *n* = 200).

Discharge-Time Cutoff	*n* Prolonged/*n* Total	OR for NOD2 Variant (95% CI)	*p*
>24 h (primary analysis; Table 6)	100/200	14.55 (3.06–69.04)	<0.001
>48 h	69/200	2.89 (0.90–9.27)	0.075
>72 h	62/200	1.99 (0.64–6.16)	0.235

All models adjusted for stricture-passage failure, age, and log-transformed CRP and IL-6, using Firth’s penalized (bias-reduced) logistic regression; only the NOD2 estimate is shown for brevity. CRP remained a significant independent predictor (*p* < 0.001) at every cutoff. Stricture-passage failure was also significant at the >48 h and >72 h cutoffs (*p* = 0.0004 and *p* < 0.001, respectively) but not at the >24 h cutoff (*p* = 0.099; Table 6), likely reflecting the near-complete separation between stricture-passage failure and the outcome at that specific threshold, which widens the confidence interval despite the large point estimate. OR: odds ratio; CI: confidence interval.

## Data Availability

The data supporting the findings of this study are available from the corresponding author upon reasonable request.

## Abbreviations

NOD2	Nucleotide-binding oligomerization domain containing 2
LDLT	Living donor liver transplantation
ERCP	Endoscopic retrograde cholangiopancreatography
WBC	White blood cell
CRP	C-reactive protein
T.Bil	Total bilirubin
ALP	Alkaline phosphatase
GGT	Gamma-glutamyl transferase
BUN	Blood urea nitrogen
PT	Prothrombin time
INR	The international normalized ratio
